# Intraductal Papillary Mucinous Neoplasms in Hereditary Cancer Syndromes

**DOI:** 10.3390/biomedicines10071475

**Published:** 2022-06-22

**Authors:** Devarshi R. Ardeshna, Shiva Rangwani, Troy Cao, Timothy M. Pawlik, Peter P. Stanich, Somashekar G. Krishna

**Affiliations:** 1 Department of Internal Medicine, Ohio State University Wexner Medical Center, Columbus, OH 43210, USA; devarshi.ardeshna@osumc.edu (D.R.A.); shiva.rangwani@osumc.edu (S.R.); 2 College of Medicine, Ohio State University, Columbus, OH 43210, USA; troy.cao@osumc.edu; 3 Department of Surgery, The Ohio State University Wexner Medical Center, Columbus, OH 43210, USA; timothy.pawlik@osumc.edu; 4 Division of Gastroenterology, Hepatology and Nutrition, Department of Internal Medicine, The Ohio State University Wexner Medical Center, Columbus, OH 43210, USA; peter.stanich@osumc.edu

**Keywords:** intraductal papillary mucinous neoplasms, hereditary intraductal papillary mucinous neoplasms, hereditary cancer syndromes, pancreatic ductal adenocarcinoma, familial pancreatic cancer, pancreatic cancer screening

## Abstract

Hereditary pancreatic cancer, which includes patients with familial pancreatic cancer (FPC) and hereditary pancreatic cancer syndromes, accounts for about 10% of all pancreatic cancer diagnoses. The early detection of pre-cancerous pancreatic cysts has increasingly become a focus of interest in recent years as a potential avenue to lower pancreatic cancer incidence and mortality. Intraductal papillary mucinous cystic neoplasms (IPMNs) are recognized precursor lesions of pancreatic cancer. IPMNs have high prevalence in patients with hereditary pancreatic cancer and their relatives. While various somatic mutations have been identified in IPMNs, certain germline mutations associated with hereditary cancer syndromes have also been identified in IPMNs, suggesting a role in their formation. While the significance for the higher prevalence of IPMNs or similar germline mutations in these high-risk patients remain unclear, IPMNs do represent pre-malignant lesions that need close surveillance. This review summarizes the available literature on the incidence and prevalence of IPMNs in inherited genetic predisposition syndromes and FPC and speculates if IPMN and pancreatic cancer surveillance in these high-risk individuals needs to change.

## 1. Introduction

Pancreatic cancer is one of the leading causes of cancer-related deaths worldwide with a 5-year survival of less than 10% [1]. Dismal survival among these patients is related to late diagnosis and lack of effective treatment [2]. Somatic and germline genetic mutations have been implicated in pancreatic cancer development. Activating gene mutations in oncogene *KRAS* and inactivating mutations in tumor suppressor genes *TP53*, *CDKN2A* and *SMAD4* play a crucial role in the formation of pancreatic cancer [3]. Germline mutations in genes associated with hereditary cancer syndromes account for up to 10% of “heritable” pancreatic cancer [4,5,6].

Heritable pancreatic cancer can be broadly divided into hereditary pancreatic cancer syndromes and familial pancreatic cancer (FPC). Pancreatic cancers arising in patients with inherited genetic predisposition (hereditary breast and ovarian cancer syndrome, Lynch syndrome, Peutz Jeghers syndrome) that increases the risk of pancreatic cancer are defined as the hereditary pancreatic cancer syndromes. The underlying mutations for heritable pancreatic cancer syndromes are predominantly inherited in autosomal dominant patterns and affect the next generation with 50% probability. Therefore, such patients have a high risk of developing pancreatic cancer and need committed pancreatic cancer screening. In FPC, pancreatic cancers arise in patients with strong family history of pancreatic cancer (at least two first-degree relatives (FDRs)) without any known genetic predisposition syndromes. The risk of pancreatic cancer in FPC increases with more affected blood relatives.

Pancreatic cancer screening through the International Cancer of the Pancreas Screening (CAPS) Consortium guidelines, American Gastroenterological Association (AGA) guidelines and the American College of Gastroenterology guidelines (ACG) is designed to detect pancreatic cancer early and improve survival in such high-risk patients [7,8,9]. Almost 85% of patients with pancreatic ductal adenocarcinoma (PDAC) have locally advanced or metastatic disease at diagnosis [10]. About 15% of pancreatic cancers arise from intraductal papillary mucinous neoplasms (IPMNs) [11,12,13]. Therefore, the early detection of pre-cancerous pancreatic cysts through screening has gained intense focus in recent years to potentially lower PDAC-associated mortality.

Pancreatic cancer screening has revealed a high prevalence of IPMNs in high-risk populations; among patients with FPC, 10–18% patients had IPMNs [14,15]. Finding a potentially pre-malignant lesion in patients at high risk of cancers is anxiety provoking. Due to the lack of data on the natural progression of these IPMNs or their relationship with IPMNs in the general population, their management is provider dependent. Consequently, some providers have a very low threshold for surgery. A meta-analysis reported that two out of three pancreatic surgeries in patients undergoing screening did not have high-risk lesions or cancer [16]. Understanding the natural progression of IPMNs in such high-risk patients is necessary to develop evidence-based management guidelines. The first step towards this end is to determine the incidence and prevalence of IPMNs in such patient population [17]. This review summarizes the available literature on the incidence and prevalence of IPMNs in inherited genetic predisposition syndromes and FPC and speculates if IPMN and pancreatic cancer surveillance in these high-risk individuals needs to change.

## 2. Origins of PDAC

PDAC comprises the majority of all pancreatic cancers and arises from the exocrine pancreatic ductal cells. There are five precursor lesions of PDAC (Table 1): pancreatic intraepithelial neoplasias (PanINs), IPMNs, mucinous cystic neoplasms (MCNs), intraductal oncocytic papillary neoplasms (IOPNs) and intraductal tubulopapillary neoplasms (ITPNs) [18,19]. PanINs are small (<1 cm) epithelial neoplasms within the pancreatic duct with a variable degree of atypia [20,21]. All PanINs have *KRAS* mutations, with *CDKN2A/p16* mutations often found in high-grade PanIN lesions [22,23,24]. Due to their small size, PanINs are not identified via radiological modalities and are thus not ideal targets for PDAC screening.

MCNs and IPMNs are >1 cm lesions, often incidentally found during cross-sectional imaging. These lesions are ideal for PDAC screening, and multiple guidelines on pancreatic cystic lesions characterize them into high- and low-risk cysts to guide management [26,27,28,29,30]. IPMNs are papillary projections of mucin-producing epithelium in the main pancreatic duct (main-duct IPMNs) or its branches (branch-duct IPMNs). Main-duct IPMNs and branch-duct IPMNs have 36–100% and 6–50% risk of developing PDAC, respectively [31]. The development of PDACs within IPMNs has been hypothesized to occur in one of three ways [32]:

(i) Sequential pathway—sequential acquisition of driver and tumor suppressor gene mutations in IPMNs leading to PDACs;
(ii) Branch-off pathway—after acquisition of key early genetic alterations, PDAC acquires new mutations not present in the IPMN;
(iii) De novo pathway—Distinct PDAC that has unique mutations compared with the concomitant IPMN.

Evidence for sequential and branch-off pathways is derived from the presence of similar genetic mutations in IPMNs and PDACs (Table 2) [33]. Several studies have reported that while *KRAS* and *GNAS* are highly specific for early IPMNs, the accumulation of other pathogenic variants in a stepwise and straightforward fashion may transform IPMNs into PDAC [34,35,36,37]. Additionally, PDACs that arise from sequential or branch-off pathways have worse prognosis than the de novo PDACs [38].

Although *GNAS* and *KRAS* mutations are highly specific and sensitive for the diagnosis of IPMNs [47], there is substantial genetic heterogeneity among various driver mutations, and these mutations can exist within the same lesion [46,48]. Additionally, low-grade IPMNs have more genetic heterogeneity than IPMNs that have high-grade dysplasia [49]. IPMNs are multifocal, and 20% of patients have recurrence or new disease after resection [50,51,52,53]. This “field defect” of the pancreas that IPMNs herald along with high genetic heterogeneity suggests that the genetic pathway from IPMNs to PDAC is more complex than previously hypothesized. MCNs are unifocal mucinous lesions that lack the risk of recurrence; thus, no PDAC surveillance is necessary after surgical resection.

ITPNs are >1 cm intraductal nodular masses with minimal cystic formation or mucin production and are associated with PDAC in more than 50% of the cases [54,55]. These are rare and account for only few overall PDAC cases [56]. IOPNs are >1 cm cystic nodular lesions with oncocytic features and ductal differentiation and are associated with PDAC in 60% of the cases [57,58]. IOPNs are newly defined, and their significance in PDAC is still being elucidated.

## 3. Pancreatic Cancer Screening

The United States Preventative Services Task Force (USPSTF) guidelines do not recommend pancreatic cancer screening in the general population with average risk of pancreatic cancer [59]. However, high-risk individuals and patients with strong family history of pancreatic cancer require screening. The CAPS Consortium [7], AGA [8] and the ACG [9] have published guidelines on pancreatic cancer screening in such individuals (Table 3). The CAPS Consortium, AGA and ACG guidelines recommend screening patients with Peutz Jeghers syndrome and Familial Atypical Multiple Mole Melanoma (FAMMM), regardless of family history of pancreatic cancer. Both AGA and ACG guidelines also recommend pancreatic cancer screening in patients with Hereditary Pancreatitis regardless of family history of pancreatic cancer. All three guidelines recommend pancreatic cancer screening in BRCA2, PALB2, ATM and Lynch syndrome patients with at least one affected FDR. The National Comprehensive Cancer Network (NCCN) has similar guidelines for cancer screening in patients with hereditary genetic predisposition syndromes [60,61].

Among patients without such hereditary genetic predisposition syndromes, the guidelines differ in their recommendations. AGA guidelines recommend pancreatic cancer screening for patients with two affected blood relatives. The CAPS Consortium guidelines recommend pancreatic cancer screening among patients with two affected blood relatives provided one is an FDR. The ACG recommends cancer screening among patients with one affected FDR. These differences in cancer screening for FPC patients may be due to lack of data on FPC as described below. Screening should occur through endoscopic ultrasound (EUS), magnetic resonance imaging (MRI) or computed tomography (CT) at different ages depending on the underlying risk.

Overall, all pancreatic cancer screening guidelines focus only on family history of pancreatic cancer and omit history of IPMNs in family members. However, 42% patients with PDAC do not have a family history of PDAC and would be missed by these guidelines [62]. IPMNs, the potential pre-cursor lesions to PDAC, are more prevalent among patients with a history of hereditary genetic predisposition syndromes and individuals with FPC. While “familial” IPMNs have not been previously described, cases of hereditary IPMNs (affected mother and son) without any history of underlying extra-pancreatic malignancy or defined hereditary genetic predisposition syndrome have been reported [63]. While these data might not provide enough evidence, such findings suggest that perhaps hereditary genetic predisposition syndromes are related to IPMNs rather than PDAC. In turn, the cumulative genetic alterations in IPMNs may increase the risk of PDAC among such high-risk patients.

## 4. IPMNs in FPC

All pancreatic cancer screening guidelines recognize a strong family history of pancreatic cancer in the risk assessment for patients. The risk of pancreatic cancer increases by 4.6-, 6.4- and 32-fold for one, two and three or more affected FDRs, respectively [64]. The causative genetic mutations of FPC are still unknown. Numerous studies evaluating the efficacy of screening methods (endoscopic ultrasound, computer tomography, magnetic resonance imaging (MRI), endoscopic retrograde cholangiopancreatography) have utilized high-risk populations such as FPC patients. These studies have often noted a higher prevalence of IPMNs and other pancreas abnormalities such as cysts, duct dilations and nodules rather than pancreatic cancer (Table 4). Overall, these studies report IPMN incidence to be about 2-4-fold higher than that of pancreatic cancer.

Three studies of all the reports listed in Table 4 merit attention (Canto et al. 2002, Canto et al. 2006, and Ludwig et al. 2010) [14,65,66]. These studies had <10% non-FPC patients in their patient population, while the other reports included >30% of subjects with hereditary genetic predisposition syndrome. It is important to note that these three studies collectively report that the incidence of IPMNs is more than twice the incidence of pancreatic cancer among FPC patients. Currently, there is a lack of data purely focused on FPC patients. Additionally, Canto et al. 2006 reported that one-half of IPMNs detected on repeat screening had previous pancreatic abnormalities identified during initial screening [14]. Biopsy of an IPMN revealed in situ PDAC validating the sequential pathway of PDAC formation.

**Table 4:** The incidence of IPMNs and pancreatic cancer among patients with significant family history of pancreatic cancer.

	HRI	Total	IPMNs	PC
Canto et al. 2002 [65]	FPC or PJS	38	1 (PJS)	1 (FPC)
Canto et al. 2006 [14]	FPC or PJS	78 (72 FPC + 6 PJS) 149 controls	7 1 cyst in control	2
Poley et al. 2009 [67]	FPC or genetic predisposition syndromes	45 (13 FAMMM + 21 FPC + 3 HP + 2 PJS + 3 BRCA1 + 2 BRCA2 + 1 p53)	7	2
Verna et al. 2010 [68]	3 BRs or 2 FDRs or 2 BRs with 1 FDR or genetic predisposition syndrome	24	3	2
Ludwig et al. 2011 [66]	1 FDR or 2 BRs or BRCA with FH of PC	109	5	1 (FDR)
Al-Sukhni et al. 2012 [69]	2 FPCs or genetic predisposition syndromes or HP or FDR of double primary cancer patient	262 (159 FPC + 7 PJS + 68 BRCA2 + 11 p16 + 5 BRCA1 + 2 HP + 10 double primary)	15 (9 FPC, 4 BRCA2, 1 HP, 1 double primary)	3 (2 FPC and 1 BRCA2)
Sud et al. 2014 [70]	2 FDRs or 3 BRs or HP or PJS or p16 or Lynch with FH of PC	16	1	2
Chang et al. 2017 [71]	Any BR	303	47	7/18 (pathological diagnosis)
Gangi et al. 2018 [72]	2 BRs (including 1 FDR) or PJS or HP or FAMMM or BRCA2 mutations with FH	58 (48 ≥ 1 FDR + 9 BRCA2 + 1 PJS)	1 (2 FDR)	0

HRI—high-risk individuals; IPMNs—intraductal papillary mucinous neoplasm; PC—pancreatic cancer; FPC—familial pancreatic cancer; PJS—Peutz Jeghers syndrome; BR—blood relative; FDR—first-degree relative; FH—family history; HP—hereditary pancreatitis; FAMMM—familial atypical multiple mole melanoma syndrome.

## 5. IPMNs in Hereditary Genetic Predisposition Syndromes

Along with FPC, certain hereditary genetic predisposition syndromes confer higher risk of pancreatic cancer in patients. Various case reports have identified IPMNs in patients with polycystic kidney disease [73,74], Lady Windermere Syndrome [75], parathyroid adenoma concerning for multiple endocrine neoplasia type 1 or type 2A, [76] cystic fibrosis [77], BRCA2 [78] and Lynch syndrome [79]. The underlying mutations in IPMNs were not analyzed in these case reports. Thus, the presence of IPMNs may be coincidental. Such case reports did not shed light on the prevalence of IPMNs or allowed a comparison to the prevalence of PDAC in these patients to be performed. Other studies have analyzed mutations in IPMNs and extrapancreatic malignancy among patients with hereditary syndromes and have reported similar genetic alterations (Table 5). A multivariate analysis revealed that germline mutations among patients with hereditary cancer syndromes was a predictor of the presence of IPMNs (relative risk, 3.2; 95% confidence interval (CI): 1.6–6.4) independent of family history of pancreatic cancer (*p* = 0.22) [80]. These data suggest that hereditary syndromes may predispose patients to IPMNs, which, in turn, increases the risk of PDAC formation.

### 5.1. McCune Albright Syndrome (MAS)

MAS is a rare, autosomal dominant syndrome resulting from an activating *GNAS* mutation. It presents with polycystic fibrous dysplasia, precocious puberty and cafe au lait spots. Genetic studies of IPMNs have noted that somatic *GNAS* mutations play an important role in IPMN formation. A total of 54 patients with MAS underwent contrast-enhanced MRI and magnetic resonance cholangiopancreatography (MRCP); IPMNs were identified in 25 (46%) patients [82]. The mean age of patients who had an IPMN detected was much lower, at 35.1 years, in this patient population than the mean age of 63 years in the general population [96,97]. Ten patients had worrisome or high-risk features; of note, among two patients who underwent surgical resection, one individual (age: 27 years) had a high-grade IPMN. These data suggest that underlying pathogenic germline *GNAS* mutations potentially increase the risk of IPMNs with advanced neoplasia in patients with MAS. In another study of 29 patients with MAS who underwent MRI/MRCP, 3 (16%) patients had an IPMN (mean age 27 years) [81]; one 50-year old patient with MAS had PDAC within an IPMN [83].

### 5.2. Lynch Syndrome/Hereditary Non-Polyposis Colorectal Cancer

Lynch syndrome is an autosomal dominant hereditary syndrome resulting from germline mutations in mismatch repair (MMR) genes (*MLH1*, *MSH2*, *MSH6, PMS2* and *EPCAM*) that predispose patients to various malignancies, especially colon and endometrial cancer [98]. The relative risk of developing pancreatic cancer is from 9.0 to 11.0 for patients with these mutations [88,99]. Among 445 patients with PDAC, 9 patients had MMR gene deficiency and 3 of these patients had Lynch syndrome according to Bethesda criteria [100]. The study also reported that MMR deficiency occurred at a lower frequency in sporadic PDAC versus IPMN-related PDAC (1.6% vs. 6.9%, *p* = 0.02) [100]. Genetic analyses of IPMNs in Lynch syndrome patients have detected mutations in *MSH2* and *MSH6* [84,85,86]. While MMR gene deficiency occurs at a low frequency in IPMNs in the general population, the presence of similar MMR gene mutations in IPMNs and Lynch syndrome among these patients suggests that Lynch syndrome may activate IPMN formation, leading to PDAC formation [101].

### 5.3. Peutz Jeghers Syndrome (PJS)

PJS is an autosomal dominant hereditary syndrome characterized by mucocutaneous pigmentation and numerous gastrointestinal hamartomas. It is linked to germline mutations in the *STK11* gene [102,103,104]. This mutation has been associated with an independent increase in the relative risk of 132 (95% CI: 44–261) for PDAC; as such, patients with PJS require screening regardless of family history [90]. A comparative mutational analysis of 22 IPMNs among patients with and without PJS demonstrated that *STK11*/*LKB1* mutations were present in 100% (2/2) of samples from PJS patients versus only 25% (5/20) of samples from patients without PJS [89]. These data suggest that *STK11*/*LKB1* mutation plays a role in IPMN formation. A molecular analysis of PDAC revealed that the loss of heterozygosity in tumor suppressor gene *STK11*/*LKB1* plays an important role in both sporadic and familial pancreatic cancer [105]. Given the higher risk of PDAC in PJS and similar mutations in IPMNs and pancreatic cancer, *STK11/LKB1* mutations noted in IPMNs among PJS patients may predispose this patient population to higher rates of progression to PDAC versus the general population.

### 5.4. Hereditary Breast and Ovarian Cancer Syndrome (HBOC)

*BRCA1* and *BRCA2* are tumor suppressor genes that code for a protein responsible for the repair of breaks in DNA strands [106]. Mutations in the *BRCA2* gene predispose patients to breast and ovarian cancer and confer a relative risk from 3.0 to 9.0 to develop pancreatic cancer [107]. The pathogenesis of *BRCA2* gene mutations that lead to PDAC remains unclear. Pancreatic cancer patients with a family history of pancreatic cancer have a higher prevalence of *BRCA2* gene mutation than pancreatic cancer patients without a family history of pancreatic cancer (17–19% [108,109] vs. 5–10% [110,111], respectively). Screening all *BRCA2* patients demonstrated a 17% prevalence of IPMNs compared with 1% in the general population [112]. The same study reported a 4% prevalence of PDAC among *BRCA2* patients, which was considerably lower than the prevalence of IPMNs.

Similar to *BRCA2*, *BRCA1* mutations also increase the risk of pancreatic cancer (relative risk, 2.26; 95% CI: 1.26–4.06) [113]. When patients with a *BRCA1* mutation were screened using cross-sectional imaging, 8.3% (3/36) of individuals had an IPMN [112]. While both *BRCA1* and *BRCA2* mutations have been extensively studied relative to pancreatic cancer risk, these screening studies have also demonstrated a higher prevalence of IPMNs in patients with *BRCA* mutations.

### 5.5. Familial Atypical Multiple Mole Melanoma (FAMMM)

FAMMM is an autosomal dominant syndrome associated with a germline mutation in *CDKN2A* gene *p16-Leiden* resulting in familial melanomas and multiple atypical nevi. The *CDKN2A p16-Leiden* gene mutation predisposes patients to PDAC with a relative risk from 13.0 to 39.0 [114,115,116,117]. Among 19 families with FAMMM syndrome, 15 patients from 7 families had pancreatic cancer versus 0 patients from 8 families without FAMMM syndrome [114]. In a recent study, 77 patients with the *p16-Leiden* germline mutation were screened using MRI/MRCP, and 9% (7/77) had pancreatic cancer, while 11% (9/77) had cystic lesions that were not otherwise categorized [118]. Overall, FAMMM patients have a 17% cumulative risk of developing pancreatic cancer by age 75 [114]. The higher prevalence of IPMNs compared with pancreatic cancer among FAMMM patients suggests that the high risk of pancreatic cancer may be mediated through increased IPMN formation from the *p16-Leiden* mutation.

### 5.6. Familial Adenomatosis Polyposis (FAP)

FAP is an autosomal dominant hereditary syndrome secondary to germline mutations of the adenomatous polyposis coli (*APC*) gene resulting in early colonic carcinogenesis. *APC* gene mutation confers a relative risk of 4.46 (95% CI 1.2–11.4) to develop pancreatic cancer [119]. Genetic analyses of IPMNs among FAP patients have reported an absence of the *APC* protein that is typical of the adenomas in FAP patients elsewhere [91,92,93]. This body of evidence suggests that *APC* mutations that transform normal mucosa to adenomas to colon cancer may also play a role in increasing the risk of PDAC through mutations in IPMNs.

### 5.7. Carney Complex (CNC)

CNC is a hereditary syndrome that increases the risk of various tumors due to an alteration in the *PRKAR1A* gene mutation. In a CNC patient registry with 354 patients, 3 patients had an IPMN with a *PRKAR1A* mutation and 1 patient had PDAC [95]. This mutation has not typically been identified in IPMNs and may suggest a potential pathway for increased risk of PDAC in CNC patients.

## 6. Summary

IPMNs have an estimated prevalence of ~5% in the general population [120]. While previous single-center studies have suggested similar rates of IPMNs in the general population and high-risk patients, the current review suggests IPMN prevalence to be much higher among patients with hereditary genetic predisposition syndromes and FPC [17,121]. This is likely explained by a detection bias, since hereditary pancreatic cancer patients undergo more frequent imaging surveillance. Alternatively, the higher prevalence of pre-malignant lesions and pancreatic cancer in hereditary pancreatic cancer patients could be explained by a direct tumorigenesis pathway encompassing IPMNs and pancreatic cancer. One recent study reported that, compared with the general population, IPMNs among patients with FPC had shorter duration of progression to PDAC. This progression persisted despite the absence of traditional risk factors such as smoking, obesity, alcohol consumption and diabetes [122]. Nearly half of the high-risk individuals who have no pancreatic lesions may develop a neoplastic lesion in 11 months, and about half of high-risk individuals with prior known lesions may show rapid growth with progression beyond the pancreas within 21 months [123]. However, more studies are needed to corroborate these findings. There is growing evidence that IPMNs are the result of a neoplastic “field-defect” involving the entire ductal epithelium of the pancreas [124]. Mutations associated with hereditary pancreatic cancers may be contributing to such field-defect, resulting in IPMNs in some foci of the ductal epithelium while generating pancreatic cancer at another focus. Moreover, some of the higher-risk IPMNs may also progress to pancreatic cancer through the sequential pathway. Continued research is essential to better understand the prevalence and progression of IPMNs to pancreatic cancer in these patient populations; in the meantime, providers should continue to closely monitor high-risk patients as per current guidelines [7,8,9].

## Figures and Tables

**Table 1:** Summary of pre-cursor lesions.

Lesion	Somatic Mutations [13,18,20,23,25]	Size	Risk of Development into PDAC	Prevalence of Progression to PDAC [16,17]
PanIN	*KRAS, CDKN2A/p(16) (High Grade)*	<1 cm	<1% (Low grade), 40% (High grade)	~80%
IPMN	*KRAS, GNAS, CDKN2A/p16, TP53, SMAD4*	>1 cm	6–50% (Branch duct), 36–100% (Main duct)	15%
MCN	*ATM/GL13*	>1 cm	<1%	5–10%
IOPN	*PRKACA, PRKACB*	>1 cm	60%	<1%
ITPN	*-*	>1 cm	50%	<1%

PanIN—pancreatic intraepithelial neoplasia; IPMN—intraductal papillary mucinous cystic neoplasm; MCN—mucinous cystic neoplasm; IOPN—intraductal oncocytic papillary neoplasm; ITPN—intraductal tubulopapillary neoplasm.

**Table 2:** Prevalence of somatic mutations in IPMNs that are typically found in PDACs.

Genetic Mutation	Prevalence in IPMNs
*KRAS*	30–80% [39,40]
*GNAS*	40–79% [25,37,41,42]
*RNF43*	14–38% [25,37,42,43]
*CDKN2A/p16*, *TP53*, *SMAD4*	40% [44,45]
*GLI3*	28% [46] (Combined prevalence in IPMNs/MCNs)
*ATM*	17% [46] (Combined prevalence in IPMNs/MCNs)

IPMN—intraductal papillary mucinous neoplasm; MCN—mucinous cystic neoplasm.

**Table 3:** Screening guidelines for hereditary pancreatic cancer patients according to the International Cancer of the Pancreas Screening (CAPS) Consortium guidelines, American Gastroenterological Association (AGA) guidelines and the American College of Gastroenterology (ACG) guidelines [7,8,9].

Hereditary Genetic Predisposition Syndromes	Affected Family Members		
	No FH	1 FDR	2 BR
Peutz Jeghers syndrome	CAPS/AGA/ACG		
FAMMM (CDKN2A/p16)	CAPS/AGA/ACG		
Hereditary pancreatitis (PRSS1)	AGA/ACG		
BRCA2		CAPS/AGA/ACG	
BRCA1		AGA/ACG	
PALB2		CAPS/AGA/ACG	
ATM		CAPS/AGA/ACG	
Lynch syndrome		CAPS/AGA/ACG	
None		ACG	CAPS */AGA

* Includes at least one first-degree relative (FH—family history; FDR—first-degree relative; BR—blood relative).

**Table 5:** Prevalence/incidence of IPMNs and pancreatic cancer in hereditary genetic predisposition syndromes.

	Gene Mutation	IPMNs	PC
MAS	*GNAS*	16–46% [81,82]	1 case [83]
Lynch	*MLH1*, *MSH2*, *MSH6*	3 cases [84,85,86]	0.7–3.7% [87,88]
PJS	*STK11*/*LKB1*	100% [89]	11–36% [90]
FAP	*APC*, *MUTYH*	3 cases [91,92,93]	3% (4/127 including 2 endocrine carcinomas, 1 acinar cell carcinoma, 1 pancreatoblastoma) [94]
CNC	*PRKAR1A*	0.8% [95]	1.7% (6/354 with only 1 PDAC) [95]

MAS—McCune Albright syndrome; PJS—Peutz Jeghers syndrome; FAP—familial adenomatosis polyposis; CNC—Carney complex syndrome; IPMNs—intraductal papillary mucinous neoplasms; PC—pancreatic cancer; PDAC—pancreatic ductal adenocarcinoma.
